# Effect of subanesthetic dose of esketamine on postoperative rehabilitation in elderly patients undergoing hip arthroplasty

**DOI:** 10.1186/s13018-023-03728-2

**Published:** 2023-04-03

**Authors:** Menghang Min, Chengxu Du, Xiaoying Chen, Wenqi Xin

**Affiliations:** Department of Anesthesiology, Huaihe Hospital, Henan University, 8 Baobei Road, Gulou District, Kaifeng, 475000 China

**Keywords:** Esketamine, Femoral neck fracture, Elderly patients, Total hip arthroplasty, Anxiety, Depression, The fascia iliaca block

## Abstract

**Objective:**

The majority of individuals with femoral neck fractures opt for total hip replacement to enhance their quality of life. However, this group frequently exhibits perioperative symptoms of pain, anxiety, and sadness, which extends recovery time to some extent. Esketamine, the right-handed monomer of ketamine, is more popular these days due to its sedative, analgesic, and antidepressant properties. There are currently few domestic and international research on the use of esketamine in elderly individuals who have undergone surgery for a femoral neck fracture. In order to further cut the length of the hospital stay and hasten postoperative recovery, this study investigates whether esketamine postoperative analgesia can lessen postoperative pain, anxiety, and depression in older patients having hip replacement.

**Methods:**

150 patients, ASA physical status I–II, aged ≥ 60 years, no limitation in gender, BMI 18–25 kg/cm^2^, who underwent selective total hip arthroplasty, according to random number table method, esketamine group (group A) and sufentanil group (group B) were randomized, 75 patients in each group. The two groups received general anesthesia method. At the end of the operation, PCIA was connected for analgesia. In group A, esketamine 2.5 mg/kg was mixed with normal saline to 100 ml. In group B, sufentanil 2.5 ug/kg was mixed with normal saline to 100 ml. Record the VAS scores after operation. Record the first ambulation time, ambulation distance and Patient-controlled Analgesia compression times after operation. The incidence of postoperative adverse reactions such as drowsiness, dizziness, nausea and vomiting, multilingual were recorded. ELISA was used to detect IL-6 and CRP in the morning, 24 h and 72 h after operation. The Hospital Anxiety and Depression Scale (HAD) score and Harris score at 3 days, 1 week and 1 month after operation were followed up.

**Results:**

There was no significant difference in VAS score and PCA compression times (*P* > 0.05), but the incidence of nausea, vomiting and dizziness in group B was higher than that in group A (*P* < 0.05). Compared with group B, the levels of IL-6 and CRP in group A at 24 h and 72 h after operation were significantly decreased (*P* < 0.05). Postoperative ambulation time and ambulation distance in group A were better than those in group B (*P* < 0.05). The HAD score of group A was lower than that of group B at 3 days and 1 week after operation (*P* < 0.05). However, there was no significant difference between the two groups at 1 month after operation (*P* > 0.05). The Harris score of group A was higher than that of group B at 3 days, 1 week and 1 month after operation (*P* < 0.05).

**Conclusions:**

Esketamine can reduce short-term postoperative anxiety and depression, relieve postoperative pain and stress response, shorten bed rest time after total hip replacement, and accelerate postoperative recovery.

## Introduction

Femoral neck fractures account for roughly 54% of hip fractures [1] and are a frequent fracture type in older people [2]. Its prevalence has greatly increased as the population ages. Total hip arthroplasty is frequently utilized as the recommended treatment to enhance quality of life. These individuals, however, frequently have underlying illnesses of varying severity and a poor pain threshold [3]. Additionally, a number of stressors, including the fracture itself and surgery, may result in perioperative anxiety and depression symptoms [4], which have a significant impact on the prognosis of patients. Anesthesiologists face a pressing challenge in selecting a medication that will effectively relieve pain while also lowering anxiety and despair. As an NMDA receptor antagonist and anesthetic, ketamine also functions as sedation and analgesia [5]. Ketamine has been shown in studies to immediately alleviate the signs and symptoms of severe depression [6]. Esketamine, the enantiomer of ketamine, has a higher affinity for the NMDA receptor and may be more potently antidepressant. In this study, the effects of postoperative analgesia on the rehabilitation of elderly patients following total hip arthroplasty were examined. Esketamine and the conventional analgesic medication sufentanil were compared. The report is as follows.

## Materials and methods

### Clinical data

Since there is no accurate result of esketamine in reducing the depression rate after THA in patients with femoral neck fracture, the main measure was defined as the depression rate 7 days after surgery, assuming that the difference between the two groups was up to 20%. According to a unilateral test conducted using the PASS15.0 software, test level *α* = 0.025, 1−*β* = 0.8 inspection efficiency, and a lost to follow-up rate of 10%, it is determined that each group should contain at least 66 participants. 150 eligible patients were enrolled in the study.

The hospital’s ethics committee granted approval for this study (Ethics No.: 2022188), and patients signed informed consent forms. Patients with ASA I or II grade, age ≧ 60 years old, no restriction on gender, BMI 18–25 kg/cm^2^, without mental illness, and ability to participate with questionnaire survey were chosen at random from August 2021 to August 2022. Communication difficulties, drug test contraindications, allergies, drug misuse history, defective coagulation function, need for spinal anesthetic, recent significant setbacks, and postoperative cognitive impairment are among the exclusion criteria. They were split into two groups, one for esketamine (group A) and the other for sufentanil (group B), each with 75 cases. During the follow-up period, 8 people in the esketamine group and 10 people in the sufentanil group were lost to follow-up. In the end, 65 members of group B and 67 members of group A were counted in the data statistics. The assignments were kept a secret from the patients, anesthesiologists, and follow-up staff until the follow-up was finished.

### Methods

All patients were fasting and drinking water before operation. After entering the operating room, ECG, SBP, DBP, SPO2 and BIS were monitored. Conventional open venous access was used for intravenous infusion of compound sodium chloride injection, low flow nasal oxygen inhalation (2L/min), radial artery puncture and catheterization under ultrasound guidance, and ART was invasive monitored.

The same induction regimen was utilized for all patient groups: midazolam 0.03 mg/kg, sufentanil 0.4 ug/kg, etomidate 0.3 mg/kg, and rocuronium 0.6 mg/kg were administered intravenously. After 3 min of pressure mask oxygenation to exclude nitrogen, the laryngeal mask was applied. Once the laryngeal mask was in a good position (*V*_T_ 8–12 ml/kg and respiratory rate 10–12 times/min), mechanical ventilation was started. Propofol (4–12 mg/kg/h) and remifentanil (0.1–0.2 ug/kg/min) were infused intravenously in both groups to maintain anesthesia, and sevoflurane (0.6–1%) was used for inhalation anesthesia. The anesthetic dosage was modified based on the procedure and level of anesthesia, and rocuronium was administered 20 mg at a time based on the duration of the procedure. Maintain PETCO_2_ at 35–45 mmHg while keeping the BIS value between 40 and 60. Sufentanil 5–10 ug was administered, or the concentration of inhaled anesthetic medications was adequately adjusted and the fluid velocity was managed, if heart rate and blood pressure during the procedure were more than 20% of the normal value. Rapid fluid replacement should be administered first, and the depth of anesthetic should be suitably decreased, if the blood pressure is less than 20% of the baseline value. Vasoactive medications should be used sensibly if the outcome is unsatisfactory, such as a 1 mg intravenous injection of methoxamine. Ten minutes prior to the completion of the procedure, sevoflurane and propofol were terminated, and 5 ug of sufentanil was administered intravenously. When the procedure was finished, remifentanil pumping speed was adjusted, and drug pumping was stopped at the end of surgery. Observe the patient's urine volume during anesthesia to maintain volume balance.

The two groups performed an ultrasond-guided lateral fascia ilicaca block utilizing the suprainguinal facial iliaca (iliaca) technique, 0.33% ropivacaine + 0.66% lidocaine 30 ml. At the same time, PCIA was connected for analgesia. Combined with the references and preliminary experimental results, Esketamine (Jiangsu Hengrui Pharmaceutical Co., LTD., specification: 2 ml/50 mg, batch number: 210922BL), dissolved in normal saline and prepared to 100 ml, was given to group A at a dose of 2.5 mg/kg, with a background dose of 2 ml/h, a PCA dose of 3 ml, and a locking period of 15 min. Sufentanil (Yichang Renfu Pharmaceutical Co., LTD., specification: 1 ml/50ug, batch number: 21A03271) was dissolved in normal saline at a rate of 2.5ug/kg and then combined to a final volume of 100 ml in group B. The PCIA parameters matched those in group A.

After the procedure, the patient was transferred to the Postanesthesia Care Unit (PACU), where the laryngeal mask was removed once the patient's spontaneous breathing and level of consciousness had returned to normal. After 30 min of observation, the patient was sent back to the ward when the vital signs stabilized and the Steward score reached > 4. PCA compression is advised to ease pain if the pain score is > 4 after returning to the ward.

### Observational indices

Patients' demographic information was gathered, including age, gender, BMI, level of education, occupation, chronic illness, preoperative HAD and preoperative Harris scores, length of surgery, and length of hospital stay. The first time out of bed, the ambulation distance, and the number of PCA presses within 48 h after operation were also noted. The resting VAS score at 2, 4, 12, 24 and 48 h after the operation and the active VAS score at 12, 24 and 48 h after the operation were also noted. The incidence of postoperative adverse reactions such as drowsiness, dizziness, nausea and vomiting, multilingual were recorded. In the morning of the surgery day (*T*0), 24 h (*T*1), and 72 h (*T*2) after the procedure, 5 ml of venous blood were taken from the patients.and the serum was separated using a centrifuge (Changsha Weikang Xingying Centrifuge Co., LTD.) for 10 min at 3000 rpm. Using an ELISA kit, the serum levels of Interleukin-6(IL-6) and C-reactive Protein(CRP) were detected (Wuhan Finn Biotechnology Co., LTD.). At 3 days, 1 week, and 1 month following surgery, the Harris score and The Hospital Anxiety and Depression Scale (HAD) scores were monitored.

### Statistical analysis

SPSS26.0 software was adopted for data analysis, measurement data are represented as $\overline{x}$ ± s, independent sample *t*-test was used for data comparison between groups, and *M*(*Q*1, *Q*3) was used for data not in accordance with normal distribution, Mann–Whitney test was used, measurement data were used for $\chi^{2}$ test, *P* < 0.05 indicated a statistical significant.

## Results

### General data

There was no significant difference in baseline characteristics between the two groups (*P* > 0.05), as shown in Table 1.

**Table 1:** Comparison of general data between the two groups

	Group A (*n* = 67)	Group B (*n* = 65)	*t*/$\chi^{2}$	*p*
Age($\overline{x}$ ± s, years)	73.9 ± 6.5	73.9 ± 6.1	− 0.012	0.990
Gender(M/F, *n*)	32/35	30/35	0.034	0.853
BMI($\overline{x}$ ± s, kg/m^2^)	22.2 ± 1.3	22.0 ± 1.3	1.220	0.225
ASA(I/II, *n*)	28/39	23/42	0.571	0.450
Chronic diseases(with/without, *n*)	37/30	40/25	0.541	0.462
Hospital costs($\overline{x}$ ± s, RMB)	30,489.5 ± 2861.6	30,012.2 ± 2958.5	0.942	0.348
The operation time($\overline{x}$ ± s, min)	117.8 ± 12.1	120.0 ± 13.6	− 0.987	0.325
Occupation(with/without, *n*)	25/42	28/37	0.456	0.499
Eduration level(Below junior high/Junior high and above, *n*)	31/36	33/32	0.268	0.605
Preoperative HAD score of anxiety($\overline{x}$ ± s, points)	9.2 ± 2.0	9.6 ± 2.0	− 1.089	0.278
Preoperative HAD score of depression($\overline{x}$ ± s, points**)**	9.6 ± 1.8	9.1 ± 1.9	1.577	0.117
Preoperative Harris score($\overline{x}$ ± s, points**)**	29.8 ± 3.1	29.5 ± 2.8	0.640	0.523

### Postoperative VAS score

There was no significant difference in VAS scores between the two groups at rest and in active state after operation (*P* > 0.05), which were comparable (Table 2).

**Table 2:** Comparison of postoperative VAS scores between the two groups (points, M(IQR))

Group	VAS score at rest					VAS score in active		
	post-op 2 h	Post-op 4 h	Post-op 12 h	Post-op 24 h	Post-op 48 h	Post-op 12 h	Post-op 24 h	Post-op 48 h
Group A	2.0 (1.0~3.0)	3.0 (2.0~3.0)	3.0 (2.0~3.0)	2.0 (1.0~3.0)	1.0 (1.0~2.0)	5.0 (4.0~6.0)	6.0 (5.0~7.0)	5.0 (4.0~6.0)
Group B	2.0 (2.0~3.0)	3.0 (2.0~3.0)	3.0 (2.0~4.0)	2.0 (2.0~3.0)	1.0 (1.0~2.0)	5.0 (5.0~6.0)	6.0 (5.0~7.0)	5.0 (4.0~6.0)
z	− 0.594	− 0.797	− 0.857	− 1.701	− 1.582	− 1.069	− 0.033	− 0.275
*p*	0.553	0.425	0.392	0.089	0.114	0.285	0.973	0.783

### Patient recovery, length of hospital time and PCA compression times

**Fig. 1:**
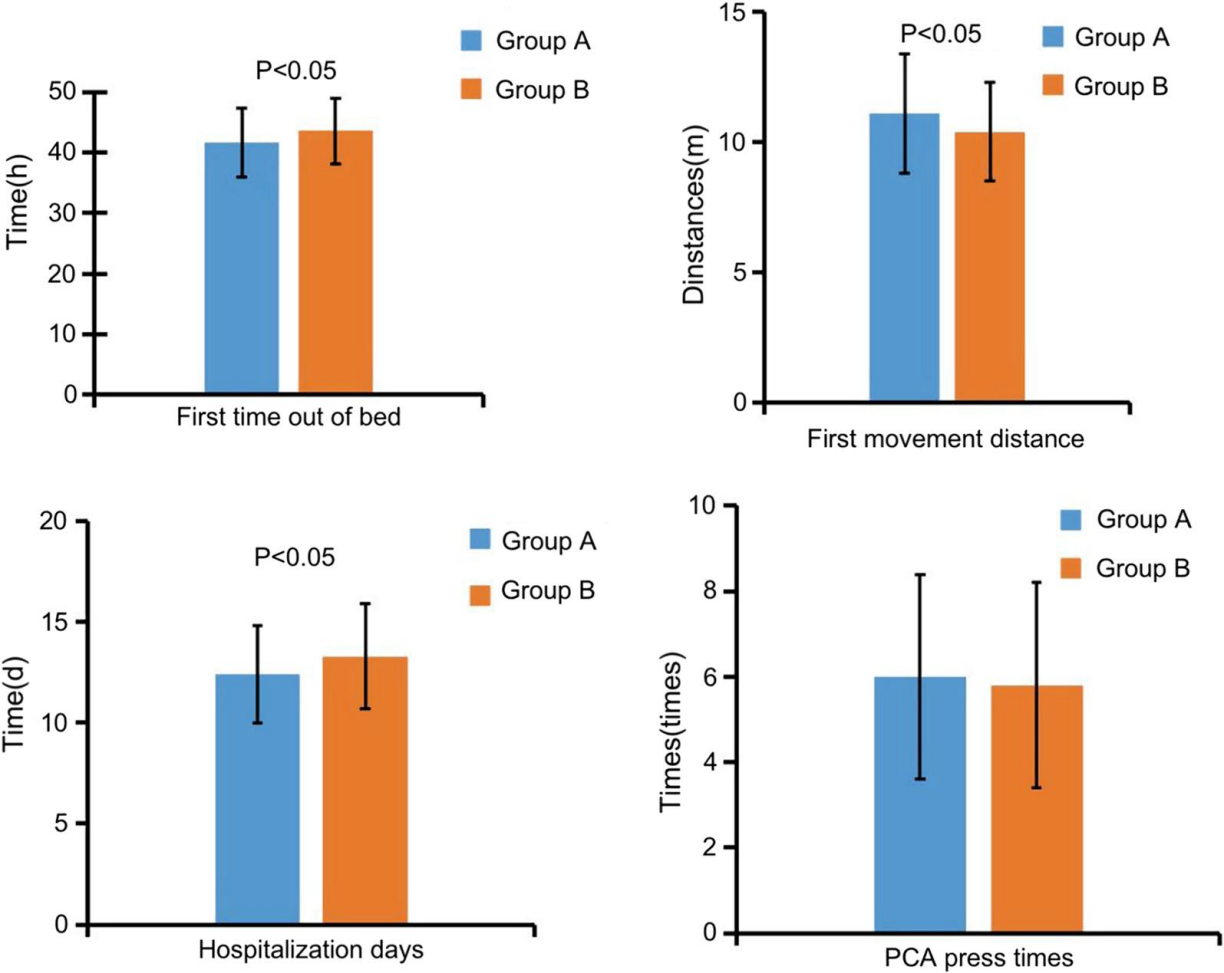
The first time to get out of bed, the distance of activity and the length of hospital stay were compared between the two groups, *P* < 0.05; PCA compression times were compared between the two groups, *P* > 0.05

The time of first getting out of bed, the distance of activity and the length of hospital stay in group A were better than those in group B, and the differences were statistically significant (*P* < 0.05). There was no significant difference in PCA compression times between the two groups (*P* > 0.05), as shown in Fig. 1.

### Comparison of adverse responses between the two groups

Compared with group A, the incidences of nausea, vomiting and dizziness in group B were significantly higher (*P* < 0.05), while there was no significant difference in the incidences of skin pruritus and multilingualism between the two groups (*P* > 0.05), as shown in Table 3.

**Table 3:** Comparison of postoperative adverse reactions between the two groups [*n* (%)]

Group	Nausea and vomiting	Itchy skin	Dizzy	Talking more than usual
Group A (*n* = 67)	10 (15)	5 (7)	8 (12)	7 (11)
Group B (*n* = 65)	26 (40)	8 (12)	18 (28)	4 (6)
$\chi^{2}$	13.971	0.872	5.176	0.796
*p*	*P* < 0.001	0.350	0.023	0.372

### Patient stress index

**Fig. 2:**
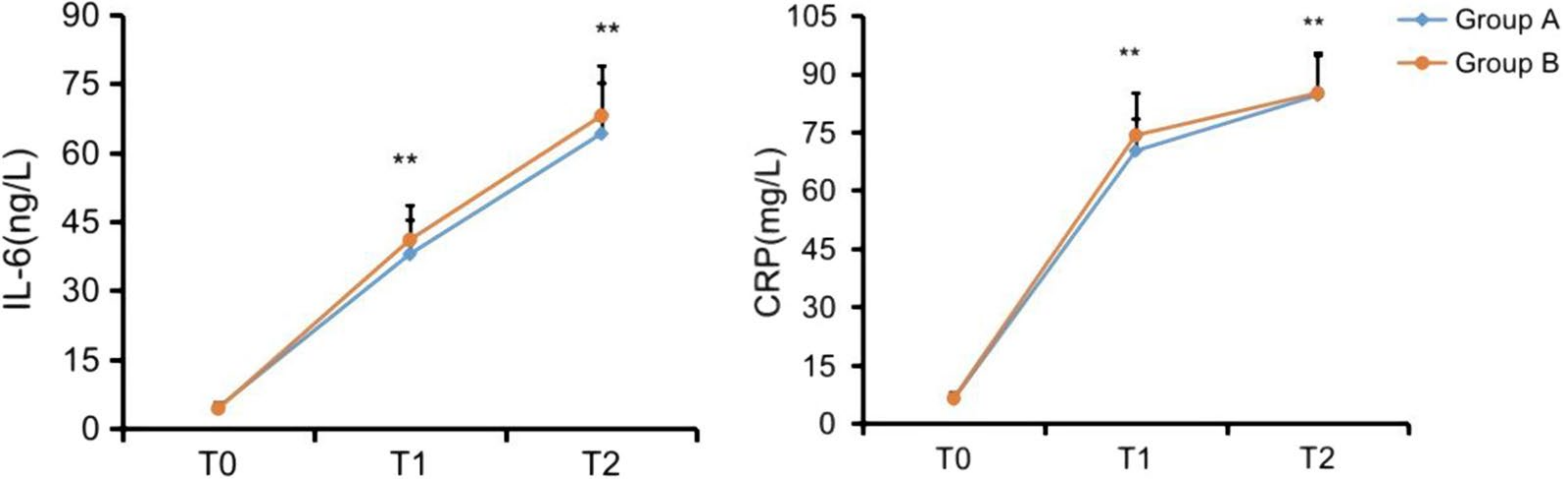
Comparison of IL-6 and CRP between the two groups. *Note*: T0 time point, *P* > 0.05; Compared with *T*1 and *T*2, *P* < 0.05

There was no significant difference in the levels of IL-6 and CRP between the two groups in the morning of the operation day (*P* > 0.05), but at 24 h and 72 h after operation, the levels of IL-6 and CRP in group A were significantly decreased (*P* < 0.05). This indicated that group A was more conducive to reducing perioperative stress response than group B (Fig. 2).

### Comparison of patients’ HAD scores

**Fig. 3:**
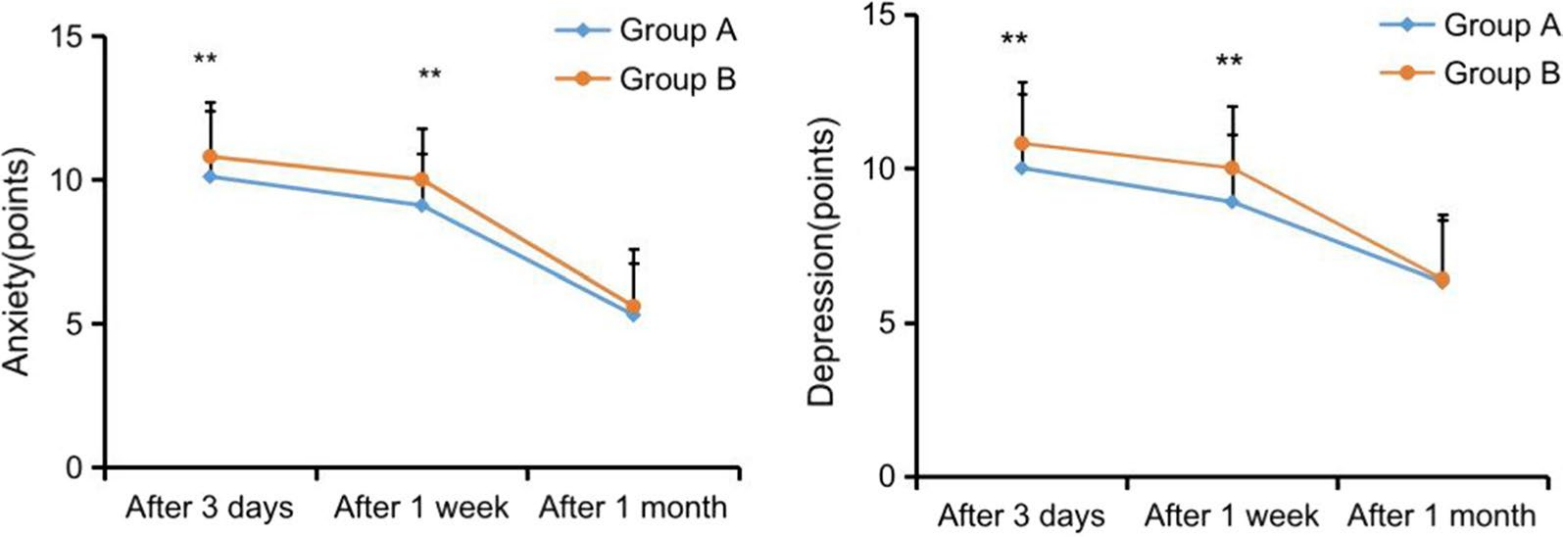
HAD scores were compared between the two groups. *Note*: 3 days and 1 week after operation, *P* < 0.05. At 1 month after operation, *P* > 0.05

Compared with group B, the scores of anxiety and depression in group A were significantly reduced at 3 days and 1 week after operation, and the differences were statistically significant (*P* < 0.05). The scores of anxiety and depression in group A at 1 month after operation showed no statistically significant difference (*P* > 0.05), as shown in Fig. 3.

### Harris score

**Fig. 4:**
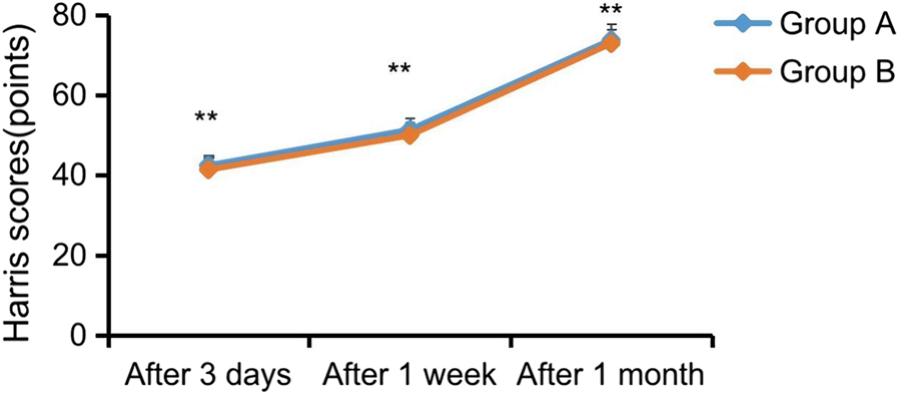
Harris score was compared between the two groups at 3 days, 1 week and 1 month after operation (*P* < 0.05)

The Harris score of group A at 3 days, 1 week and 1 month after operation was significantly higher than that of group B, and the difference was statistically significant (*P* < 0.05), as shown in Fig. 4.

## Discussion

The most common form of depression in older patients with femoral neck fractures is anxiety [7, 8], due to the fact that these patients must spend a lot of time in bed while experiencing pain from the fracture and mistrust of their prosthetic joints. Pain is one of the most prominent contributing variables among the common adverse events following THA [9]. Perioperative pain and anxiety and depression are risk factors for each other, and this association between them has long been established [10]. Increased subjective pain perception is a result of depression and anxiety, and emotional problems are also a result of chronic acute pain. The accompanying depressive feelings will progressively go away after the pain is reduced [11]. Esketamine, a brand-new ketamine medication, has more potent analgesic and antidepressant properties than ketamine. Esketamine was initially authorized by the US Food and Drug Administration for the treatment of resistant depression on March 4, 2019. An intravenous injection of 0.2 or 0.4 mg/kg of esketamine could produce a rapid and potent antidepressant effect within 40 min, according to Singh [12] et al. double-blind’s randomized controlled study on patients with refractory depression. The antidepressant effect was better in the low-dose group. Esketamine was chosen as the research subject for this study because, according to the consensus guide for the intravenous treatment of acute pain with ketamine [13], it can be administered to the greatest number of patients with the greatest benefit and the least risk when used for postoperative analgesia. The guide also states that, in the absence of stronger supervision, ketamine should not be administered in doses greater than 1 mg/kg/h per hour. Because there is no specific recommendation for the postoperative analgesic dose of esketamine, research has shown that esketamine play efficiency is 2 times that of ketamine [14], and in the case of acute pain patients who cannot tolerate high doses, can use low doses (0.1–0.5 mg/kg/h) to achieve analgesia effect. Accordingly, we employed a low dose of 1/2 of ketamine, through the preliminary test, found that can reach the optimal analgesic effect. Following THA, the fascia ilicaca block is a popular analgesic technique that can significantly lower the postoperative analgesic score and speed up patient recovery. The study’s control medication was sufentanil, a conventional opioid analgesic. We discovered that 50 ug of sufentanil was similar to 50 mg of esketamine based on prior studies [15, 16]. In order to maintain the rigorousness of the test, the dosage of sufentanil chosen for this investigation was chosen to be equivalent to the impact of esketamine.

The results of this study showed that there was no significant difference in VAS score between the two groups within 48 h after operation, but in terms of adverse effects, esketamine group in the incidence of nausea and vomiting and dizziness than sufentanil group. In a meta-analysis on postoperative deopioid analgesia published by Wang et al. [17], the results showed that perioperative intravenous infusion of esketamine did not increase the risk of postoperative nausea and vomiting, but increase the incidence of psychiatric adverse reactions which was mainly related to the reduced use of opioids. It has been confirmed in literature that continued use of opioids after surgery can increase the incidence of nausea and vomiting [18]. Although in the past, ketamine can cause a series of negative consequences, including hallucinations, nightmares, delirium, cognitive impairment and so on, esketamine as its right-lateral monomer, effectively reduced the number of symptoms associated with anesthesia, in this study, there were 7 cases of multilingual symptoms in group A, which was more than that in group B, but the last two groups were not statistically significant.

At the same time, surgery, as a kind of trauma treatment, is also a major source of stress after surgery, in which inflammatory factors will also change significantly under the stress of surgery, while IL-6 and CRP, often as stress indicators of pain, have also been proved to play an important role in the diagnosis and treatment of depression, which is considered to induce immune response in the human body under stress [19]. Haapakoski et al. [20] found that patients with depressive symptoms generally had higher levels of IL-6 and CRP. In this study, the postoperative follow-up revealed that group A's HAD score was lower than group B's at 3 days following surgery and remained lower for 1 week. Additionally, group A’s stress index was statistically significantly lower than group B’s at 24 and 72 h following surgery, indicating that esketamine was superior to sufentanil not just in terms of analgesia. Esketamine has also been demonstrated to lessen the symptoms of transient depression. One month after surgery, the effects of esketamine had worn off and the patient’s body had other components that were masking the antidepressant benefits.

When total hip replacement patients were followed up for almost 3 years, Schwartz et al. [21] discovered that the depression group had a worse prognosis, including overmedication, surgical problems, an extended hospital stay, and even readmission for revision therapy. Additionally, negative emotions experienced by patients can result in sympathetic nerve excitation, personality changes, and communication issues with family members and medical professionals, all of which can have an impact on patients’ postoperative recovery. These factors include stress, postoperative pain, and activity restrictions following hip surgery. The results of this study showed that patients in group A recovered more quickly with reduced anxiety and sadness than those in group B, and that this improvement was extended to the first time they got out of bed, the distance they had to go to get out of bed, and the Harris hip score.

## Conclusions

Esketamine has good clinical application value because it can more effectively relieve pain in elderly patients who have had total hip arthroplasty, reduce perioperative stress response, improve perioperative anxiety and depression symptoms, shorten bed rest time, and encourage postoperative rehabilitation.

## Abbreviations

THA: Total hip arthroplasty
VAS: Visual analog score
HAD: Visual analog score
SBP: Systolic blood pressure
DBP: Diastolic blood pressure
ART: Arterial blood pressure
ELISA: Enzyme-linked immunosorbent assay
NMDA: *N*-methyl-d-aspartic
PCIA: Patient-controlled intravenous anagesia
